# Advancements in 3D Printing and Nanomaterials for Personalized Drug Delivery

**DOI:** 10.2174/0115672018420806251204083146

**Published:** 2026-01-22

**Authors:** Arjun Gokulan M., Narayanan J.

**Affiliations:** 1 Department of Pharmacology, SRM College of Pharmacy, Faculty of Medicine and Health Sciences, SRM Institute of Science and Technology, Kattankulathur, Chengalpattu- 603 203, Tamilnadu, India

**Keywords:** 3D printing, nanomaterials, drug delivery, bioprinting, implants, personalized medicine, FDM, stereolithography

## Abstract

3D printing, also known as additive manufacturing, has transformed drug delivery by enabling the development of complex, patient-specific dosage forms and implantable systems tailored to individual therapeutic needs. This review explores the convergence of 3D printing technologies with nanomaterials in the fabrication of advanced drug delivery systems and biomedical implants. Key 3D printing techniques, including Fused Deposition Modeling (FDM), Stereolithography (SLA), Direct Energy Deposition (DED), and electrospinning, are discussed alongside their material compatibilities, such as polymers, metals, ceramics, and composites. Nanomaterials-like dendrimers, liposomes, polymeric nanoparticles, carbon nanotubes, and exosomes-are critically examined for their roles in enhancing drug stability, targeted delivery, and controlled release. The paper highlights innovative drug delivery strategies, including polypills, gastro-floating tablets, and compartmentalized dosage systems, enabled by precise 3D printing. Additionally, recent advancements in 3D-printed drug-eluting implants for localized therapy in cancer and infectious diseases are presented. These systems demonstrate prolonged release profiles, biocompatibility, and mechanical properties resembling those of human tissue. Despite scaling and regulatory challenges, the future of this technology lies in the integration of smart materials, surface-modified nanoparticles, and AI-assisted design, paving the way for decentralized, personalized, and sustainable medical solutions.

## 1 INTRODUCTION

Digital fabrication is a trending process that uses computer-controlled tools and machines to produce physical objects from digital designs [1]. Computer-Aided Design (CAD) is well defined for creating 2D and 3D models of objects [2]. 3D printing is a fast-emerging technology that can create physical objects from a geometrical representation by successive addition of materials [1]. It has become a significant part of modern manufacturing. 3D printing builds parts layer by layer using an additive approach; hence, it is also known as an additive technique, rapid prototyping (RP), additive manufacturing (AM), or additive process [3]. In 1986, Charles W. Hull designed and patented the first-ever 3D printer, based on stereolithography, in which objects are created through the solidification of layers of photopolymer (resin) [4]. Later, in 1989, S. Scott and Lisa Crump invented another model based on the fused deposition modeling (FDM) technique, in which a nozzle moves along the three axes of the printer to deposit molten material layer by layer to produce a final object [5]. They also co-founded Stratasys, Ltd., which feeds a plastic filament into a heated extruder and precisely lays the material. When the key patents expired, this led to the RepRap movement. Bowyer and his team developed and designed parts of the 3D printer, naming it the RepRap project, with printers named Darwin and Mendel [6]. Since then, 3D printing has experienced tremendous growth, and being open-source, it has expanded into many fields, including research, design, and manufacturing [7].

In recent years, 3D printing has evolved from simple prototyping to more complex, application-specific manufacturing. It has flourished into the advancement in the 3 different stages. In the initial stages, 3D printing was common among architects, artists, and designers. It was used to make only the sample models of their ideas. Many startups and established companies use 3D printing today to produce prototypes before mass production. The 3D printing edged the making of prototypes by various advantages, such as being easy to replicate and remodel (the product can be facsimiled and adjusted as needed), economically affordable (in comparison to the classical approach to designing the prototype, 3D printing is a cheaper approach), and security and privacy (it is easy to make prototypes without any involvement of a mediator) [8, 9]. Several industries have benefited from 3D printing; for example, a shoe manufacturer can create hundreds of models in just a few hours [9]. Many other industries also take advantage of 3D printing for prototype design, saving several days compared to traditional methods [10]. Another benefit is the availability of cheaper raw materials; for instance, if the final product is to be manufactured in metal, the prototype can be printed using plastic or resin. This reduces the cost of expensive inputs and saves time [11]. As 3D printing technology advanced, it progressed from being primarily used for prototypes to being employed in final production. This technological leap has enabled significant advancements in biomedical applications, including the fabrication of custom implants, drug delivery devices, and tissue scaffolds tailored to individual patient needs. This phase was known to be direct digital manufacturing or rapid tooling [12]. During this period, 3D printing was used in bridge manufacturing and for producing small batches of products before final production. It proved particularly useful in test marketing, allowing the printing of various product sizes, shapes, and models to present a wide range of options for customers [13]. Terry Wohlers, a well-known industry expert, stated that 20% of 3D-printed products are final products rather than prototypes. By this time, 3D printing had evolved rapidly, becoming accessible to the general public. People were able to use 3D printers much like ordinary printers, making it possible to download a digital file and print a product at home instead of purchasing it from a store.

Building on these foundations, 3D printing has rapidly expanded beyond industrial prototyping into critical sectors such as healthcare. One of the most transformative applications is in the development of personalized drug delivery systems. Coupled with advances in nanomaterials, 3D printing now enables the creation of smart, patient-specific therapeutic solutions. These innovations offer enhanced precision, targeted delivery, and improved treatment efficacy, marking a significant shift in modern pharmaceutical and biomedical research. It was used in house décor and in creating decorative parts for gas stoves, appliances, or furniture. It was also used in fabricating the innovative toys, action figures, and games [14].

## 2 METHODOLOGY

### 2.1 Search Strategy

The search was conducted using a combination of keywords and Boolean operators: Keywords: “3D printing,” “additive manufacturing,” “nanomaterials,” “drug delivery,” “personalized medicine,” “bioprinting,” “drug-eluting implants,” “smart drug delivery systems,” Boolean operators: (“3D printing” OR “additive manufacturing”) AND (“nanomaterials” OR “nanocarriers”) AND (“drug delivery” OR “personalized medicine”).

### 2.2 Inclusion Criteria

- Articles published in English;
- Studies focusing on 3D printing technologies, nanomaterials, and their integration in personalized drug delivery systems;
- Research articles, systematic reviews, meta-analyses, and conference proceedings.

### 2.3 Exclusion Criteria

- Non-English articles;
- Studies unrelated to pharmaceutical applications;
- Editorials, commentaries, and papers lacking sufficient experimental or clinical relevance.

### 2.4 Study Selection and Data Extraction

Two independent reviewers screened the retrieved articles based on the title and abstract, followed by full-text evaluation. The following data were extracted:

- 3D printing techniques and materials used;
- Types of nanomaterials applied;
- Applications in drug delivery and personalized therapy;
- Advantages, limitations, and regulatory considerations.

### 2.5 Data Synthesis

The extracted information was synthesized qualitatively and organized thematically into sections covering 3D printing techniques, nanomaterials, drug delivery applications, biomedical implants, future perspectives, and regulatory considerations.

## 3 TYPES OF 3D-PRINTING

### 3.1 Binder Jetting

Binder jetting is a multi-purpose 3D printing technology used to generate complex parts by selectively depositing binding agents onto a powder bed. Binding agents selectively join powder particles, layer by layer, forming complex structures without the need for support.[1]. The top layer of spread powder is formed using the jet chemical binder [2]. This process requires materials such as gypsum-based powder and water-based binders. Other various binders and materials can be used, and it is also used to design coloured parts. This process is more desirable than powder-bed fusion techniques. In this process, there is no necessity for supporting structures, and it also allows the recycling of unused powders. It offers rapid parts production, fewer material limitations, and high-quality products in metal and ceramic materials [15]. The binder jetting process is used to build casting patterns, raw sintered products, or similar large-volume products from sand. Metals, sand, polymers, hybrids, and ceramics can also be printed using this technique. Some materials used in the process do not require additional processing, like sand. Large products can also be printed using this method. Therefore, the binder jetting method is fast, cheap, and simple, as powdered particles are fixed together. This method has been explored in pharmaceutical applications to create porous scaffolds for tissue engineering and drug-loaded tablets, owing to its ability to fabricate complex geometries without support structures.

### 3.2 Material Jetting

Photopolymers and wax are used in material jetting to print different materials together and to design supporting structures for various applications [1]. Droplets of photopolymers are solidified layer by layer using UV light to create detailed, multi-material structures. The viscosity of the droplets is a critical parameter for successful processing; high viscosity can make the layers difficult to flow, while low viscosity may promote excessive flow, potentially resulting in unwanted deposition of multiple layers on top of each other. Particle deformity can be minimized through proper solidification and by increasing material strength. This process enables the creation of high-surface-quality products with complex, detailed parts and precise dimensions. However, post-processing can affect surface quality. The printed products can also be coloured, and this colouring process is relatively inexpensive and straightforward [7]. Materials used in material jetting include polymers, ceramics, composites, biologicals, and hybrids [2]. This technique has been applied in the pharmaceutical field for printing dosage forms with tailored drug combinations, particularly due to its high resolution and multi-material capability.

### 3.3 Direct Energy Deposition

Direct Energy Deposition (DED) is a complex printing technique used to repair or add material to existing components. It produces high-quality objects with a greater degree of control over the product’s structure. In this method, similar to material extrusion, the nozzle can move in multiple directions. DED can be applied to various materials, including ceramics, polymers, metals, and metal-based hybrids such as wire or powder. Laser deposition and Laser-Engineered Net Shaping (LENS) are technology-based principles of DED that can generate or repair products ranging from millimeters to meters. Due to its scalability and versatility, DED has been adopted in sectors such as tooling, transportation, and aerospace [16]. Although it is more commonly used in orthopaedics and dental implants, DED also holds potential in the biofabrication of customized prostheses and tissue repair constructs, where precise deposition of biomaterials is essential.

### 3.4 Powder Bed Fusion

Powder bed fusion is a fast, high-accuracy 3D printing process that uses an electron beam or laser to melt or fuse material powders. It consists of electron beam melting (EBM), Selective Laser Sintering (SLS), selective Heat Sintering (SHS), and direct metal laser sintering (DMLS) [17]. Powder bed fusion, particularly Selective Laser Sintering (SLS), has been successfully employed in producing drug-loaded oral dosage forms and biocompatible implants with precise control over porosity and mechanical strength.

#### 3.4.1 Selective Laser Sintering (SLS)

Selective Laser Sintering (SLS) is a powder-based fabrication technique developed by Carl Deckard in 1984 and patented in 1990. It is widely used in the biomedical and pharmaceutical fields for designing oral and maxillofacial prosthetics, implants, tissue engineering scaffolds, surgical tools for neurological procedures, and for fabricating physical models through selective solidification of various powders. This technique uses laser energy to selectively fuse powder particles into solid structures with high density, producing parts with near-full density and minimizing the need for extensive post-processing. SLS is classified as a powder-based fusion technique, in which a high-powered energy source sinters or partially melts a bed of powder-resin, metal, or polymer—to fuse the particles together [18]. An SLS 3D printer consists of a printing chamber and a powder reservoir, heated to a temperature just below the melting point or glass transition temperature of the material. Laser energy density, scanning speed, scanning strategy, and bed temperature are key factors influencing the mechanical properties of the product. Powder characteristics, such as morphology, granulometry, density, and flowability, are also considered during selection. The powder bed in the printing chamber acts as a support structure, allowing effective fabrication of overhanging layers. SLS is similar to Selective Laser Melting (SLM); however, in SLS, the material is sintered rather than fully melted, making it more suitable for materials with low thermal conductivity. This method can be used with polymers, metals, ceramics, and composites, depending on the powder properties and printer system. SLM, in contrast, uses a wide range of metals, including steel, titanium, nickel, magnesium, aluminum, copper, cobalt-chrome, tungsten, and gold. Advantages of SLS include relatively low production costs, the ability to control surface properties, and minimal post-processing requirements. Its limitations include longer printing times for oral solid dosage forms, difficulty in printing flat surfaces, limited accuracy, and challenges with highly complex geometries.

#### 3.4.2 Electron Beam Melting (EBM)

Electron beam melting uses a beam of electrons to melt metal powder into solid objects. The process begins with modelling the parts in 3D, sending the model to the slicer, which cuts it into layers, then loading the metal powder into the printer's tank and depositing it in thin layers. Preheat the mixture by fusing the powder with an electron beam; repeat this process until the parts are complete, and then remove the parts from the mixture. This process is used in various industries, including aerospace, automotive, medical, and defence. The Electron Beam Melting (EBM) method produces strong, dense products and is energy efficient. However, it can result in a rough surface finish and make it difficult to remove trapped powder.

#### 3.4.3 Selective Heat Sintering (SHS)

It is an additive manufacturing process. This cost-effective technique sinters thermoplastic powders using localized heating, making it suitable for small-scale, low-cost applications. It is an ideal method, as it has an inexpensive evaluation testing procedure. It uses a less intense thermal printhead than selective laser sintering. It is cheaper and scalable to desktop size [19].

#### 3.4.4 Direct Metal Laser Sintering

It is also called direct metal printing or selective laser melting. Laser energy precisely melts metal powders into solid, functional components used in dental and orthopaedic applications. It guides the laser using a computer-aided design file to melt the selected parts of powdered metal. It has certain limitations, such as a limited build volume, porous parts, high cost, and slow printing speed. It is used in various sectors, including aerospace, medical, dental, jewellery, and prototyping. It uses various metals and alloys, such as titanium, nickel-based alloys, cobalt-chrome alloys, and copper-based alloys [20].

### 3.5 Sheet Lamination Method

In this method, thin sheets of material are bonded to form an object. Laminated Object Manufacturing (LOM) and Ultrasonic Additive Manufacturing (UAM) are certain examples [2]. LOM uses adhesives to bond material layers, while UAM uses ultrasonic waves to fuse metal layers. It enables full-colour printing. It is cost-effective and can recycle the excess materials [21]. While not commonly used for drug delivery, sheet lamination holds potential for fabricating large-scale biomedical models and anatomical structures for surgical planning and education.

### 3.6 Vat Photopolymerization

In this method, a liquid photopolymer is exposed to a laser, light, or UV rays. This process mainly includes two types: Stereolithography (SLA) and Digital Light Processing (DLP). SLA uses UV-sensitive resin influenced by additives, while DLP uses conventional light sources(Pagac *et al*., 2021) [22].

#### 3.6.1 Stereolithography

Stereolithography (SLA) is a popular variant of VAT polymerisation and an essential technology in additive manufacturing (AM). It uses thermoset photopolymer materials, which undergo chemical and mechanical property changes when exposed to light. The working principle of SLA is guided by predetermined parameters, such as layer height and light source, which influence surface quality [2]. Despite producing high-quality products, SLA has certain limitations, including reduced impact resistance and potential loss of mechanical strength over time due to mechanical stress. It is, however, one of the best methods for producing complex structures, ensuring that small, intricate parts are fabricated with precise dimensions and excellent surface quality. SLA is widely used in the automotive and medical sectors, with predicted growth in future applications. It has been successfully applied in orthodontics, cardiovascular disease treatment, behavioural and tissue interaction tests, neurosurgery, spine surgery, and traumatology [23]. SLA offers the unique advantage of accurately printing difficult shapes. Its drawbacks include limited capability for multi-material printing, potential low resolution in some cases, and relatively high cost. SLA is particularly valuable for producing precise drug delivery devices, such as microneedles and patient-specific implants, due to its ability to fabricate intricate structures with high resolution.

#### 3.6.2 Digital Light Processing

It traces each layer with a laser beam. It produces the entire layer in a single pass by projecting the layer's image at once, making it easy. The resolution of the product depends on the projector's pixel density. Detailed prints are best in high pixel density. Resins of such type are typically photosensitive and harden on exposure to light. Excess resin can be reused, which reduces the waste. It is widely used in the dental, medical, jewellery, and prototyping sectors. DLP has been explored for creating customized drug-eluting devices and intricate medical implants with precise geometries suitable for patient-specific therapies.

### 3.7 Fused Deposition Modelling

Fused Deposition Modeling (FDM) is a 3D printing process that produces parts directly from a CAD model. Developed by Stratasys in the early 1990s, it involves extruding molten material through a nozzle of predetermined diameter to build layers on a heated platform. FDM is inexpensive, compact, and free of organic solvents, making it frequently used in biomaterials research. In this method, the filament-usually heated above its glass transition temperature-is deposited onto the printing platform to form successive layers. FDM is safe, versatile, and compatible with a wide range of commercial machines. However, it has certain limitations in product quality and is not widely used in medical applications. A study by Goyanes demonstrated that using different polymers and printing *via* SLA resulted in faster diffusion rates compared to FDM filaments. FDM can be applied to Transdermal Drug Delivery (TDD) systems, as it eliminates the need for solvents and can be combined with Hot-Melt Extrusion (HME) processes to produce a wide range of drug/polymer blends. Improvement in technical parameters can increase the application of FDM in TDD systems [24]. Using CAD software, a virtual model is created, then it is converted to a G-code archive, and the design is duplicated layer by layer until the entire model is achieved. Crucial variables in FDM extrusion include layer thickness, raster angle, build orientation, infill density, printing speed, and materials [25]. It offers various advantages, such as low cost, ease of polymer production, low energy and temperature requirements, good durability and accuracy, and the ability to produce thermoplastic items with complex geometries. The limitations include maintenance costs, irregular filament line sizes, high printing error rates, and poor surface finish. All of these methods have been depicted in Fig. (**1**). FDM is widely used in pharmaceutical research to fabricate personalized polypills, gastro-floating tablets, and compartmentalized dosage forms, often integrating hot-melt extrusion for solvent-free drug incorporation.

### 3.8 Electrospinning

Electrospinning is a widely used technique in biomedicine that uses an electric field to spin polymers into nanofibers. It uses an electric field to stretch and solidify the polymer solution as it passes through a spinneret, creating a charge in the polymer and producing fine fibres [26, 27]. A polymer is dissolved in a solvent to create a polymer solution [28]. A high voltage is applied to a needle attached to a syringe containing the starting material, causing charges to form at the liquid's surface and creating an intense electrical field between the needle tip and the collector. A charged jet of fluid is ejected towards the collector at a critical voltage when the repulsive electrostatic force of the liquid exceeds the liquid's surface tension. If the starting material is a solution, solvent evaporation or solidification will result in an interconnected web of nanofibers. The final fibre design may be customised by modifying the electrospinning process's processing parameters [29]. By modifying the electrospinning solution's characteristics and the process parameters, it is possible to precisely control the morphology and structure of the resulting fibres. To alter fibre composition and structure, a variety of electrospinning techniques are frequently used, including blend electrospinning, emulsion electrospinning, parallel electrospinning, coaxial electrospinning, and triaxial electrospinning [30]. These methods have been depicted in Fig. (**2**). Electrospinning plays a crucial role in biomedical applications, creating nanofiber mats for wound dressings, tissue scaffolds, and drug-loaded transdermal systems with controlled release profiles [31].

## 4 MATERIALS USED IN 3D PRINTING

3D printing requires high-quality material with proper specifications and a 3D printing device. Procedures, requirements, and material control agreements are to be established between the supplier, purchaser, and end user. Ceramics, metals, polymers, hybrids, hydrogels, and composites are key functional materials used in this technology [1].

### 4.1 Polymers

From prototyping to final structures with complex geometries, polymers are among the most widely used materials in 3D printing technology. Polymers with low melting points or in liquid states are particularly common due to their low weight, affordability, and flexibility. In Fused Deposition Modeling (FDM), successive layers of extruded thermoplastic filaments—such as PLA, ABS, PP, and PE—are deposited to build the object. In recent years, higher melting point thermoplastics, such as PEEK and PMMA, have also been increasingly used. Polymers can play an important role in biomaterials and medical device products, often as inert materials, contributing to device efficiency and providing mechanical support in orthopaedic implants [32]. Polymers used in 3D printing, such as PLA and PCL, exhibit low melting points and thermoplastic behavior, making them ideal for FDM and SLA techniques. Their biocompatibility and controlled degradation profiles enable their extensive use in biomedical implants, transdermal patches, and drug-loaded scaffolds.

### 4.2 Ceramics

Ceramics and concrete can produce 3D objects with minimal pores or cracks, resulting in strong, durable, and fire-resistant materials suitable for building and construction. Examples include alumina powder, bioactive glass, and zirconia. The mechanical strength of alumina powder can be enhanced using 3D printing technologies, expanding its applications. SLA machines can process glass-ceramics and bioactive glass into dense parts, further improving their mechanical properties. In the nuclear power sector, zirconia is valued for construction applications, including element tubing, due to its low radiation susceptibility and thermal neutron absorption [32]. Ceramics such as alumina and bioactive glass possess high compressive strength and thermal stability, making them ideal for powder-based printing methods. Additionally, their osteoconductive properties and chemical inertness make them highly suitable for bone regeneration and dental applications.

### 4.3 Metals

Metals are used across a wide range of industries, including aerospace, automotive, medical, and manufacturing. Materials like aluminium alloys, cobalt-based alloys, nickel-based alloys, stainless steels, and titanium alloys offer excellent physical properties, making them suitable for complex manufacturing. Cobalt-based alloys are primarily used in the dental sector, as they exhibit high specific stiffness, resilience, and heat-treatment resistance [33, 34]. Metals such as titanium and cobalt-chrome alloys are valued for their high tensile strength and corrosion resistance, making them suitable for laser-based printing techniques such as SLM and EBM. Their mechanical robustness and biocompatibility are crucial for load-bearing implants in orthopedic and dental surgery.

### 4.4 Composite

Composites include materials like carbon fibres and glass fibres. Due to their versatility, low weight, and alterable properties, these materials are widely revolutionised in high-performance industries [35]. Composites, particularly carbon- and glass-fiber-reinforced materials, offer customizable mechanical properties and lightweight characteristics. Their tunable stiffness and wear resistance expand their use in patient-specific prosthetics, bone plates, and structural drug delivery devices fabricated *via* extrusion-based or hybrid methods. Carbon fibres are used in aerospace due to their high specific stiffness, strength, corrosion resistance, and fatigue performance. Glass fibres have high performance and are cost-effective.

## 5 DESIGN CONSIDERATIONS FOR 3D-PRINTED DRUG DELIVERY SYSTEMS

### 5.1 General Drug Delivery System (DDS) Design Principles

Drug Delivery Systems (DDS) are designed to improve therapeutic efficacy, minimize side effects, and enhance patient compliance. A well-designed DDS considers drug properties (solubility, permeability), route of administration, release kinetics, and patient-specific variables such as age, metabolic rate, and comorbidities. 3D printing technologies allow precise control over formulation parameters, providing unique opportunities for designing responsive and patient-specific Drug Delivery Systems (DDS). DDS are primarily used to administer active pharmaceutical ingredients (APIs) to achieve a therapeutic effect in humans or animals. They ensure optimal drug absorption and distribution, thereby improving safety and efficacy by controlling the timing, rate, and target of drug release in the body. Various approaches exist for DDS, including controlled-release formulations and targeted delivery systems, in which the drug is active only at a specific site, such as cancerous cells or tissue. This 3D printing technology provides an effective solution for individuals and the complex production of oral drug delivery systems. 3D printing technology provides an alternative approach to creating a new host material and controlling the spatial distribution within a polymer composition [36]. The workflow of the 3D printed Biomaterials is shown in Fig. (**3**).

### 5.2 Controlled and Targeted Drug Delivery Systems

Controlled and targeted drug delivery systems aim to release drugs at a desired rate or to a specific site of action, enhancing therapeutic efficiency and reducing systemic exposure. Various nanocarriers and polymeric systems have been developed for these purposes. Controlled drug release is classified into liposomal, polymeric, and electromechanical types.

#### 5.2.1 Liposomal Systems

Liposomal drug delivery systems face two major drawbacks: *in vivo* instability and Reticuloendothelial System (RES) entrapment, in addition to other widely studied limitations. Polymeric controlled drug delivery systems are further classified into biodegradable, non-biodegradable, and soluble polymers, which act as drug carriers and can be administered *via* parenteral, oral, implantable, or other routes. Liposomes are spherical vesicles with lipid bilayers capable of encapsulating both hydrophilic and lipophilic drugs. They enhance drug stability, reduce toxicity, and are particularly suited for targeted delivery in cancer and inflammatory conditions.

#### 5.2.2 Polymeric Nanoparticles

Polymeric oral drug release systems are now cost-effective and improve patient quality of life by reducing the frequency of dosing. Polymeric nanoparticles, such as PLGA and PEGylated polymers, provide sustained and controlled drug release, are biodegradable, and offer versatile surface functionalization for targeted therapy.

### 5.3 Personalized Drug Delivery Systems

Personalized drug delivery systems are designed to meet the unique therapeutic needs of individual patients. 3D printing enables the fabrication of tailored dosage forms, combination therapies, and customizable release profiles**.** Polypharmacy plays a major role in medication errors due to regimen complexity. Multiple medications can lead to poor adherence and health outcomes. Polypill could be one of the best remedies for increasing patient compliance and medication adherence. 3D printing, also known as additive manufacturing, could be the best way to customize the combination of drugs in a polypill, enabling individualized dosing. Incompatibility is a serious issue to address during the manufacturing of a polypill; therefore, the problem can be resolved by separating the drugs using chemically compatible excipients or by compartmentalising each drug into different parts of the tablet. Pereira showcased the polypill containing 4 ingredients: amlodipine, rosuvastatin, indapamide, and lisinopril. This combined pill reduces the time and is cost-effective [37]. The multilayered polypill can provide multiple dosages and manage the controlled release of the drugs. The release of the drug can be altered by rearranging the layers of the drug. Maroni designed a two-compartment capsular device using FDM technology; it consisted of incompatible drugs or differing formulations. The dissolution of the drug was based on the thickness of the capsule. Christos introduced the concept of compartmentalisation: a tablet was printed containing two antidiabetic drugs, metformin and glimepiride. FDM uses drug-loaded filaments and nozzles to separate the drugs in a single tablet [38].

### 5.4 Gastro-retentive Drug Delivery Systems

Gastro-retentive systems are designed to prolong drug residence time in the stomach, thereby improving bioavailability for drugs absorbed primarily in the upper GI tract. 3D printing has enabled the development of complex floating systems and swellable matrices for this purpose. Solubility is the primary factor that affects the absorption of drugs in the gastrointestinal tract. 3D printing offers great control over the design, density, and structure of the drug delivery system [38].

#### 5.4.1 Huanbutta and Sangnim’s 3D-Printed Floating Drug Delivery System

This method is designed to formulate gastro-floating drug delivery systems using 3D printing technology. It consists of two parts: the cap and the body, both printed *via* 3D printing. The system is designed so that low-density material and a hollow structure ensure buoyancy in the stomach. The housings are typically printed with pharmaceutical-grade materials, such as hydroxypropyl methylcellulose (HPMC) or similar polymers, which are biocompatible and suitable for prolonged gastric use. Infill density and layer thickness must be optimized to maintain low density for floating while preserving structural integrity, particularly for controlled-release drugs. However, this approach has certain limitations, such as challenges in scaling up 3D printing, which may increase costs and production time [39].

#### 5.4.2 Chai et al.’s FDM-Printed Hollow Floating Tablets

The main objective of the study was to create a hollow floating tablet for gastric retention using 3D printing technology. The two primary parameters investigated were buoyancy and prolonged drug release, utilizing hydroxypropyl cellulose (HPC). Drugs were directly incorporated into the tablet during the 3D printing process. Developing advanced filaments that are compatible with a wide range of drugs and remain stable under Fused Deposition Modeling (FDM) conditions is challenging. FDM requires high temperatures, which can degrade heat-sensitive drugs. Therefore, alternative low-temperature 3D printing approaches need to be developed to enable broader drug compatibility [40].

## 6 TYPES OF NANOMATERIALS

### 6.1 3D Printing Nanomedicine

3D printing in medical research is essential to the development of cutting-edge 3D medicine. In order to produce innovative 3D drugs, researchers are directly integrating nanoparticles into 3D printing materials. Due to their ability to encapsulate different drugs, proteins, and DNA, nanocarriers hold enormous promise for drug delivery. Particle engineering and surface tailoring require an understanding of how particles interact with biological components. Systems of nanoparticles enhance therapeutic properties, prolong blood circulation, and enhance efficacy while reducg toxicity [41, 42].

The advanced biomaterials used are depicted in Table **1**.

### 6.2 Nanomaterials for Drug Delivery

Currently, numerous studies investigate the various forms of nanocarriers used in drug delivery, including liposomes, polymeric nanoparticles, and dendrimers, as illustrated in Fig. (**4**). The quality of nanocarriers is determined by the physicochemical properties of the selected materials and the preparation methods employed. Key factors influencing the stability and efficacy of drug-loaded nanocarriers include particle size, surface charge, hydrophobicity, and drug release rate. Many nanodrugs have been implemented in clinical practice for both therapeutic and diagnostic purposes, while others are under investigation in clinical studies. Additionally, nanocarriers are applied in the treatment of kidney disorders, skin diseases, brain disorders, and cancer (Mazayen *et al*., 2022) [43].

#### 6.2.1 Dendrimers

Dendrimers, discovered in 1978 by Fritz Vögtle, also reported by [44], are symmetrical, branching polymeric entities with a diameter of 3-10 nm. They can enhance drug bioavailability and improve transdermal permeability, solubility, stability, and oral bioavailability, making them potentially suitable for designing nanocarriers [45-47]. Dendrimers consist of a core, dendrons, and a functional group at the end, which influence solubility, polarity, bonding, and synthesis. They can be fully or partially complete and can be soluble in organic solvents or water [48]. Controlled Drug Delivery Systems (CDDS) have fewer adverse reactions and are more effective in treating diseases, especially when the therapeutic outcome does not match the pharmaceutical dosage [49, 50].

#### 6.2.2 Liposomes

Liposomes, introduced in 1965, are nano-sized lipid-based vesicles used for drug administration due to their site specificity, drug release, and reduced adverse effects [47, 51]. They are widely used as nanocarriers for polar and nonpolar substances due to their low toxicity, biocompatibility, and biodegradability [52]. Liposomes are divided into four groups based on shape and number of lipid bilayers and can be produced using various methods [53]. They are biologically compatible with human cells and have been approved for treating various diseases [54].

#### 6.2.3 Polymeric Nanoparticles

Polymeric Nanoparticles (PNPs) are widely used in biomedicine due to their drug-loading capacity, biodegradability, and biocompatibility. PNPs have reduced toxicity, increased drug penetration, controlled release, stability, and therapeutic efficacy. Common polymers include alginates, chitosan, dextran, gelatine, and poly (glycolic acid). Preparation methods include solvent evaporation, emulsification/solvent diffusion, nanoprecipitation, and supercritical fluid technology [55, 56].

#### 6.2.4 Carbon Nanotubes

Carbon Nanotubes (CNTs) are cylindrical allotropes of carbon with a high aspect ratio, exceptional mechanical strength, and electrical conductivity. These properties make them promising candidates for targeted drug delivery and biosensing applications. Carbon Nanotubes (CNTs) are a versatile material with excellent electrical, chemical, thermal, and mechanical properties. They are classified into Single-Walled Carbon Nanotubes (SWCNTs) with a single graphene layer and a diameter ranging from 0.4 to 3 nm and multi-walled carbon nanotubes (MWCNTs) with multiple layers. CNTs are produced using Chemical Vapour Deposition (CVD) and electrochemical conversion of CO₂ by molten salt-based electrolysis. Carbon Nanotubes (CNTs) are versatile materials with high electrical, chemical, thermal, and mechanical properties. They are used in various applications, such as photovoltaic devices (Ali *et al*., 2023) [57], sensors, and supercapacitors. CNTs are classified into Single-Walled (SWCNTs) and Multi-Walled (MWCNTs) layers. They are produced by chemical vapour deposition and by electrochemical conversion of CO₂ *via* molten-salt-based electrolysis. These methods have been used to optimise shape selectivity, reaction rate, and energy efficiency in CNT production (Dipta & Lee, 2024) [58].

#### 6.2.5 Exosomes

Exosomes are small extracellular vesicles that play a crucial role in intercellular communication. They are found in various cell types and are detectable in various bodily fluids. Exosomes are generated by inward budding of the endosomal membrane and can fuse with lysosomes, degrade, or fuse with the plasma membrane. They contain a wide range of molecules, including proteins, lipids, nucleic acids, and signalling molecules. They transfer biomolecules and signalling molecules between cells and are involved in various physiological and pathological processes. Exosomes are being extensively investigated for their biological functions and potential applications in regenerative medicine [59].

### 6.3 Surface Modification of Nanocarriers

Nano-drugs face challenges like elimination, antibody opsonisation, and drug resistance post-administration. To improve their effectiveness in therapy, surface modification can be achieved through physical or chemical means. Physical modification involves the adsorption of materials, while chemical modification involves chemical reactions with treatment agents [60]. As drug carriers, nanoparticles are constrained by several limitations, notably pH variability, immunological responses, formulation challenges, stability issues, and dosage management. To overcome these obstacles, Surface modification materials such as PEI, PEG, and chitosan can help [61]. The data from Table 1 provides a study that uses surface-modified nanomaterials over 5 years to increase their bioavailability, stability, and drug release at the targeted site.

## 7 3D-PRINTED DRUG-ELUTING IMPLANTS

### 7.1 Biomimetic Implant Design

Formulation challenges in 3DP technology, initially used for plastic models, include sensitivity to factors like high temperatures, light, and pressure. Physical and chemical compatibility with excipients is crucial. Most studies focus on proof of concept, loading medical devices with drugs, and selecting 'model drugs'. More research is needed to develop clinically tested materials. Non-biodegradable polymers are crucial ingredients in drug delivery implants (Al-Litani *et al*., 2023) [62].

### 7.2 Non-Biodegradable Drug Delivery Implants

On considering patient-specific therapy, hormones, and release times, 3DP technologies provide tailored therapies. This entails careful material selection and formulation approaches. Korelidou A *et al*. investigated the use of fused deposition modelling to fabricate reservoir-type implantable devices for sustained drug delivery. The tetracycline antibiotic was chosen as a model molecule to evaluate the technology's potential for prophylactic antimicrobial treatment after surgery. The researchers prepared biodegradable porous membranes using poly (lactic acid) and poly(caprolactone) (PCL) ratios. The implants were 3D-printed vertically and loaded with tetracycline, demonstrating that membranes with a 50/50 PLA/PCL ratio released the drug for up to 25 days. The implants also showed antimicrobial properties against E. coli and S. aureus. The membranes showing the highest drug release and antimicrobial properties. The implants were biocompatible, and the technology could be easily applied to clinical settings for localized treatment [63].

Wang Y *et al*. developed a 3D printing technique for treating osteosarcoma through personalized local chemotherapy. Cylindrical and spherical poly-L-lactic acid (PLLA) implants were designed using the stereolithography method. *In vivo* trials demonstrated that 3D-printed PLLA implants are excellent carriers for anticancer drugs, exhibiting favourable biodegradability, *in vitro* cytotoxicity, *in vitro* blood compatibility, *in vivo* subacute toxicity, and *in vivo* sensitization profiles. The implants’ anti-osteosarcoma efficacy surpasses that of traditional chemotherapy. The 3D-printed drug delivery system enables personalized local chemotherapy, multi-drug delivery, sustained long-term drug release, and reduces the need for reoperation in osteosarcoma treatment. Spherical implants are particularly advantageous for individualized dosing, multi-drug delivery, and long-term drug release. The PLLA implants are biodegradable, biocompatible, and safe. This technique has significant potential to impact clinical practice by allowing researchers to fabricate desired geometries for variable drug release kinetics, facilitate individualized pharmacotherapy, provide bone support during treatment, and reduce the cost of personalized therapies [64].

Di Luca *et al*. explore the use of 3D bioprinting to develop two layers of implantable scaffolds for breast cancer therapy by using Extrusion-based technologies. The first layer uses polymers such as Poly-ε-Caprolactone (PCL) and Chitosan (CS) and incorporates 5-Fluorouracil (5-FU), aiming to release the drug over 4 weeks. The second layer is made exclusively from PCL and loaded with Gold Nanoparticles (AuNPs) for radiation enhancement. The non-soluble scaffold offers a synergistic approach to conventional chemotherapy, optimizing drug release to patients' specific needs. The 3DP polymeric scaffolds are designed and printed using an environmentally sustainable process without organic solvents. The synergistic approach reduces side effects and increases patient compliance. The scaffolds remain stable after surgery, overcoming discomfort during device implantation. Mechanical tests show good mechanical behaviour and sustained drug release without mass loss [65].

Further studies will verify the mechanical properties and storage stability of 3D-printed implants, with a focus on implant formulation and printing parameters [65]. Han X *et al*. developed Polyetheretherketone (PEEK), a biomaterial with mechanical properties similar to bone and excellent biocompatibility, which is extensively used in dentistry and cranio-maxillofacial applications. However, PEEK lacks inherent antimicrobial activity, which may increase the risk of infection and impair osseointegration. In this study, totarol, a natural antibacterial agent, was coated on 3D-printed PEEK surfaces, and an optimal concentration balancing osteogenesis, cytocompatibility, and antibacterial activity was determined. Totarol coatings were applied at various concentrations to Fused Filament Fabrication (FFF) 3D-printed PEEK surfaces. The samples were evaluated for cytocompatibility using L929 fibroblasts and SAOS-2 osteoblasts *via* the CCK-8 assay. Scanning Electron Microscopy (SEM), live/dead staining, and crystal violet staining were used to assess antibacterial capacity. An optimal totarol concentration of approximately 10 mg/mL was identified, demonstrating that totarol-coated PEEK exhibits excellent biocompatibility and enhanced antibacterial activity, indicating considerable clinical potential as an orthopedic and dental implant. Mechanical properties of the PEEK samples were assessed using tensile, compressive, and bending tests. The results showed that the mechanical characteristics of the PEEK samples closely resemble those of human bone tissue, particularly trabecular bone. By matching the mechanical properties of PEEK to those of surrounding bone, the risks of osteonecrosis, implant loosening, and potential stress-shielding effects can be minimized [66].

Jing Z *et al*. present a new approach to treating osteosarcoma-caused bone defects using 3D-printed titanium alloy implants. The implants, which have lower elastic modulus, reduced stiffness, improved bone conduction, and stronger mechanical interlocking, can be loaded with cisplatin, a clinical first-line anti-osteosarcoma drug, within a PLGA-PEG-PLGA thermo-sensitive hydrogel. The study found that loading cisplatin reduced tumour volume by over two-thirds with negligible organ damage, achieving better anti-tumour effects while avoiding the adverse effects of systemic cisplatin delivery. The implants demonstrated acceptable long-term stability, with 8.48%–10.04% bone in-growth and 16.94%–20.53% osseointegration. The study also showed that cisplatin/hydrogel-loaded 3D-printed titanium Ti6Al4V implants are safe and effective, providing excellent anti-osteosarcoma activity and satisfactory bone repair. This strategy has significant potential for clinical translation, as 3D-printed titanium alloys and cisplatin have already been approved for clinical use [67]. In 3D printing, the choice of polymer is critical and depends on the material characteristics; drugs and polymers must be compatible to ensure proper function.

### 7.3 Biodegradable Drug Delivery Implants

The most commonly used materials in the 3D printing (3DP) industry are polymers due to their versatility and ease of use in various 3DP processes. They are abundant, sustainable, non-toxic, biocompatible, and biodegradable [68]. Picco C. J. *et al*. developed subcutaneous implants loaded with olanzapine using 3D printing. The implants were made from blends of OLZ (olanzapine), poly(caprolactone), and poly(ethylene glycol). *In vitro* release studies demonstrated sustained drug release over 200 days, with a maximum drug release of approximately 60% during this period. Three different formulations were successfully developed and manufactured using a robocasting 3D printer. Further studies, including *in vivo* testing with appropriate animal models and optimization of terminal sterilization strategies, are required to ensure the safety of these implants for human use [69]. Ferrero, C *et al*., explore the use of Ultrasound-Assisted Compression (USAC) and 3D-printing Semi-Solid Extrusion (SSE) technologies to create sustained-release drug delivery systems using a biodegradable aliphatic copolyester. The copolymer, derived from ω-pentadecalactone (PDL), 1,4-cyclohexanedimethanol (CHDM), and dimethyl succinate (DMS), has a low melting point and a random microstructure. The antibacterial agent metronidazole (MTZ) was used as a model drug. USAC and SSE techniques were used to create drug delivery devices with dense and meshed structures, with USAC exhibiting more reproducible physical properties. *In vitro* drug release studies showed prolonged and sustained drug release over 20 days after an initial burst release of metronidazole. The sustained-release period was shorter for SSE devices containing 10 wt% MTZ due to their larger, meshed structure. The study suggests that processing the aliphatic copolyester using both USAC and SSE technologies is an attractive strategy for producing biodegradable drug delivery systems for long-term metronidazole release [70]. Ding, Y. *et al*., developed a drug-eluting Bioresorbable Vascular Scaffold (BVS) using a photopolymerizable, citrate-based biomaterial and a high-precision additive manufacturing process. The drug-eluting bioresorbable vascular scaffold (BVS) can achieve a clinically relevant strut thickness of 62 μm and controlled release of the anti-restenosis drug Everolimus, compared to metallic drug-eluting stents. Polymer-based BVSs, such as Abbott's Absorb-the only BVS authorized by the US FDA-have shown lower clinical success due to higher strut thickness (157 μm), increased tissue inflammation, and a higher risk of major cardiac events. The drug-eluting BVS was successfully deployed in swine coronary arteries, demonstrating safety and efficacy for 28 days with inflammation profiles comparable to commercial metallic drug-eluting stents [71]. Utomo E. *et al*. developed a prolonged delivery system for Metronidazole (MTZ) in the form of intravaginal devices made from biodegradable and biocompatible polymers for the treatment of bacterial vaginosis, a condition caused by Gardnerella vaginalis. Currently, antibiotics like Metronidazole (MTZ) are used to treat Bacterial vaginosis, but they require frequent administration. So, the 3D printed devices were prepared using semi-solid extrusion (SSE), with different polymer layers and a copolymer of methyl vinyl ether and maleic anhydride. H-poly(caprolactone) and L-poly(caprolactone) were mixed to form homogeneous meshes and discs. MTZ was dispersed evenly, and the release of MTZ was faster in meshes than in discs. An additional layer of Gantrez™-AN119 was added for mucoadhesive properties. The devices showed *in vitro* drug release and antibacterial activity, with disc formulations sustaining MTZ release for 72 hours and up to 9 days. The adhesion forces were higher than those of previous vaginal drug delivery systems, enhancing residence time and clinical performance. The devices show potential as an alternative treatment for Bacterial vaginosis [72].

## 8 APPLICATIONS

3D printing technology has various applications in the fields of military foods, medicines, textiles, and civil engineering. When applied to food technology, it is referred to as food-layered manufacturing. A key advantage of 3D printing is its integration with computer-aided design (CAD) tools. It also enables the production of complex geometrical shapes in a shorter time. However, in certain cases, skilled personnel are required for the design process. For mass customization, this method provides an innovative alternative approach. Using this technique, complex geometrical structures can be fabricated. Consumer expectations across different age groups and lifestyles can be met in printed foods. Various characteristics, such as texture, flavour, and even nutrient content, can be modified using this method. This technique plays an important role in product customization, delivering foods faster than conventional methods. Thus, 3D food technology provides an improved approach for customized food design [73]. Beyond food applications, 3D printing has broad uses in critical care, general surgery, neurology, ophthalmology, orthopedic surgery, pulmonology, and vascular surgery. Many surgeons employ 3D printing for treatment planning, and radiologists must stay updated on these advanced techniques. Radiologists are key personnel responsible for proper imaging and interpretation in the medical field. 3D modelling provides surgeons with better visualization to analyze fractures, reduce operative time, and ultimately lower costs, leading to improved outcomes [74]. Furthermore, 3D technology has wide-ranging applications, including scientific research, art and creative design, educational visual aids, architectural and engineering models, custom device covers and enclosures, prototype parts, functional components, and mass production of end-use parts.

### 8.1 Construction

3D printing technology has widely evolved in the construction industry by enabling fast, low-cost, and sustainable building techniques. China-based Winsun Decoration Design Engineering Company utilized four large-scale 3D printers to build ten one-storey houses in just one day. 3D technology in these machines employed a cement-construction waste mixture, printing the walls layer by layer [75]. The houses measured approximately 10 metres wide and 6.6 metres high, and were constructed at a fraction of the cost required for traditional methods, demonstrating cost efficiency, time savings, reduced labour, lower construction waste, and improved safety.

### 8.2 Medicine

3D printing technology can be most significant in medicine; therefore, it can design a personalised healthcare plan [76].

#### 8.2.1 Hearing Aids

3D printing is employed in the design of hearing aids, which includes the benefit of customisation. Devices can be designed using 3D scanning facilities to specially fit individuals' ears with the utmost comfort [77].

#### 8.2.2 Bioprinters

Tissues and organs can be designed using bioprinters, which generally consist of cells and biomaterials. They are widely used for printing structures such as titanium pelvises, plastic tracheal splints, and titanium jaws. Bioprinted hearts and blood vessels, developed for research, testing, and potential transplantation, are particularly remarkable. In tissue engineering, vascular networks created using hydrogels have proven to be a breakthrough, as they can sustain printed tissues by providing nutrients and oxygen [78].

#### 8.2.3 Digital Dentistry

Denture fits are one of the most challenging designs, which is restrained by proper fits; therefore, 3D scanning proved to be an innovative technique ensuring the proper customised design. It is fast, reduces discomfort, and improves the health outcomes [79].

#### 8.2.4 Prosthetics

3D printing has made prosthetics more accessible at a low cost. These were especially designed for children, war victims, accident victims, athletes, and para-athletes(Erenstone, 2023) [80].

#### 8.2.5 Bionics

A 3D-printed bionic ear embedded with electronics was developed by researchers of Princeton and Johns Hopkins. This innovation combines tissue with electronics and could lead to advanced prosthetic senses for those with disabilities [81].

#### 8.2.6 Artificial Organs

Scientists at Heriot-Watt University have printed embryonic stem cell clusters, which could one day develop into full organs for transplantation [78].

### 8.3 Manufacturing

3D printing has advanced the manufacturing industry, particularly in rapid prototyping and custom production. It eliminates the traditional costs of tooling by printing parts directly from digital models. The two most prominent sectors using 3D printing in manufacturing are automotive and aerospace. Automotive industries utilize 3D printing for designing engine and interior components, while the aerospace industry uses it for producing lightweight components. Product customization has become a growing trend, allowing customers to modify 3D files according to their preferences and submit them for production [82].

### 8.4 Domestic Usage

3D printers are used to design decorative items, toys, and models, as well as to replace household parts. 3D printers are widely helpful in the DIY culture, which encourages creativity and self-sufficiency [83].

### 8.5 Clothing

The fashion industry is full of innovation and creativity; therefore, 3D printing plays a key role for both aesthetic and functional reasons. Designers experiment with custom-fit, intricate designs that are difficult to achieve with traditional methods [84].

### 8.6 Academic

3D printing is increasingly integrated into education across all levels. Students explore their innovation and creativity in molecular models, mechanical parts, and architectural structures. Enhance understanding through hands-on learning.

## 9 FUTURE PERSPECTIVES

3D printing has emerged as one of the most significant trends across various fields. It has a notable history in designing complex and customized parts without added cost or production delays, making it ideal for personalizing products in medicine, fashion, and consumer electronics. Additionally, 3D printing promotes sustainability by minimizing material waste and enabling on-demand manufacturing, which reduces inventory and transportation costs [85]. It accelerates the production development cycle through rapid prototyping and allows small businesses and startups to access manufacturing without large capital investments. Apart from this wide range of advantages, certain limitations make 3D printing difficult to adopt for large-scale production [86]. One major limitation is the lack of regulatory protocols and standardized quality assurance procedures, particularly in critical fields such as healthcare and aerospace, which poses a significant challenge for the future development of 3D printing.

However, the advancement of 3D printing is unmatched by other trends. Multi-material and multi-colour printing provides a foundation for expanding functional and aesthetic complexity in a growing market [87]. The concept of 4D printing in small materials highlights changes in shape and material properties over time in response to environmental stimuli. Focusing on developments in healthcare, 3D printing has progressed to bioprinting for the creation of functional tissues and organs, offering groundbreaking possibilities in regenerative medicine. Sustainability plays a key role in any field, and 3D printing emphasizes the use of biodegradable and recyclable materials to reduce environmental impact [88]. The integration of artificial intelligence (AI), machine learning, and the Internet of Things (IoT) into 3D printing enhances printer efficiency, enabling real-time monitoring, predictive maintenance, and automated quality control. Furthermore, global supply chains can benefit from decentralized and on-demand manufacturing, allowing localized production of goods, reducing dependency on centralized factories and lengthy logistics. This approach is particularly advantageous in remote areas and even in space exploration, where 3D printing could fabricate tools or components in real time [89]. Despite its potential, 3D printing faces challenges, and overcoming these barriers is essential to establish it as a foundational technology for the next industrial era, with the capacity to reshape the production of manufactured goods [90].

### 9.1 Sustainability and Eco-Friendly Materials

The future of 3D printing in medicine will focus on sustainable practices, using biodegradable, renewable, and less energy-intensive materials. Example: PLA and PCL are not only biocompatible but also environmentally friendly alternatives to petroleum-based polymers. Research into recyclable support structures and green solvents is expanding [91].

### 9.2 Smart and Responsive Printing

Smart printing integrates responsive materials and real-time sensing capabilities into 3D-printed devices. This enables adaptive drug release triggered by physiological factors such as pH, temperature, or enzyme levels. Example: Hydrogels embedded with biosensors can monitor local inflammation and modulate drug delivery accordingly [92].

### 9.3 Bioprinting and Tissue Engineering

Bioprinting involves the deposition of living cells and biomaterials to fabricate functional tissue constructs. Future directions include printing vascularized tissues, organoids, and even whole organs for transplantation. Example: Bioprinted skin grafts for burn victims and 3D-printed cartilage for joint repair are already under preclinical evaluation [93].

### 9.4 Personalized On-Demand Therapy

3D printing will enable decentralized, on-demand manufacturing of personalized medicine at hospitals or pharmacies, tailored to individual patient profiles.

Example: Custom polypills combining multiple medications with different release profiles, printed based on pharmacogenomic data.

### 9.5 Regulatory and Commercial Translation: Global Landscape and Future Directions

The successful clinical translation of 3D-printed drug delivery systems depends not only on technological innovation but also on a robust understanding of evolving regulatory frameworks worldwide. Currently, regulatory pathways for additive manufacturing in healthcare vary significantly between regions. In the United States, the Food and Drug Administration (FDA), particularly through its Center for Devices and Radiological Health (CDRH), classifies 3D-printed drug-device combinations under complex product categories. To support the safe adoption of such technologies, the FDA released its guidance document titled *Technical Considerations for Additive Manufactured Medical Devices* in 2017. This document outlines critical factors, including material selection, software control, device validation, and post-processing quality assurance. Within the European Union, regulation is governed by the Medical Device Regulation (MDR 2017/745) and European Medicines Agency (EMA) guidelines. In this context, CE marking for 3D-printed medical products requires rigorous evaluation of biocompatibility, structural integrity, sterility, and manufacturing reproducibility.

Countries like India and other emerging economies are in the process of developing formal guidelines to address the regulatory nuances associated with personalized, small-batch 3D-printed products. Emphasis is increasing on ensuring bioequivalence, sterility, safety, and reproducibility while still fostering innovation. Several ongoing efforts and global initiatives are shaping the commercial and clinical translation of 3D printing technologies. The FDA’s *3D Printing at the Point of Care (PoC)* initiative explores how hospitals and healthcare facilities can independently design and produce patient-specific devices on-site. In parallel, ISO/ASTM 52900 standards provide foundational terminology and frameworks to define quality and reproducibility in additive manufacturing, which are essential for regulatory harmonization. Furthermore, programs such as the *NIH’s 3D Print Exchange* and the FDA’s *Emerging Technology Program* are promoting open innovation, knowledge sharing, and early engagement between developers and regulators. These collaborative initiatives not only accelerate safe development but also streamline the regulatory approval process for novel 3D-printed therapeutics and devices.

### 9.6 Manufacturing Challenges and Scaling Considerations

Despite the growing promise of 3D printing and nanotechnology in personalized medicine, significant manufacturing bottlenecks hinder large-scale clinical translation. One of the primary concerns lies in batch-to-batch reproducibility, particularly for nanomaterials and bio-inks. Minor variations in nanoparticle size, surface functionalization, or polymer composition can result in substantial differences in therapeutic performance, stability, or safety profiles. Achieving uniformity across batches requires highly controlled synthesis conditions, real-time quality monitoring, and stringent post-production validation protocols. In the case of 3D-printed medical devices, scalability remains a substantial challenge. Transitioning from lab-scale to industrial-scale production introduces complexities related to equipment calibration, software standardization, and printer-to-printer variability. The layer-by-layer approach, while precise, often leads to longer production times and is difficult to synchronize across multiple production units without compromising resolution or mechanical integrity.

Moreover, the cost of scaling up remains prohibitive, particularly when using high-grade biodegradable polymers, pharmaceutical-grade excipients, or sophisticated nanocarrier systems. Cleanroom requirements, GMP-compliant production facilities, and validation studies further increase the financial and logistical burden. For instance, producing custom implants or polypills in small batches is economically viable only in high-income healthcare systems or for rare, individualized cases. Addressing these challenges will require integrated strategies that combine automation, AI-guided process control, and modular manufacturing platforms. Future advances may include smart bioprinters capable of self-correcting layer defects, AI-based material-prediction models, and hybrid systems that combine additive and subtractive manufacturing to enhance speed and precision.

## CONCLUSION

In conclusion, 3D printing has emerged as a transformative force in modern medicine, offering unprecedented precision, customization, and efficiency in drug delivery and implant fabrication. The integration of nanotechnology has further elevated its potential, enabling the development of smart, biocompatible nanocarriers and drug-eluting systems tailored to individual patient needs. From polypills to vascular scaffolds, the synergy between material science and additive manufacturing is redefining therapeutic possibilities. As the field advances, the convergence of 4D printing, artificial intelligence, and sustainable materials is poised to usher in a new era of decentralized, responsive, and intelligent healthcare solutions- extending even to remote and extraterrestrial environments.

## Figures and Tables

**Fig. (1):**
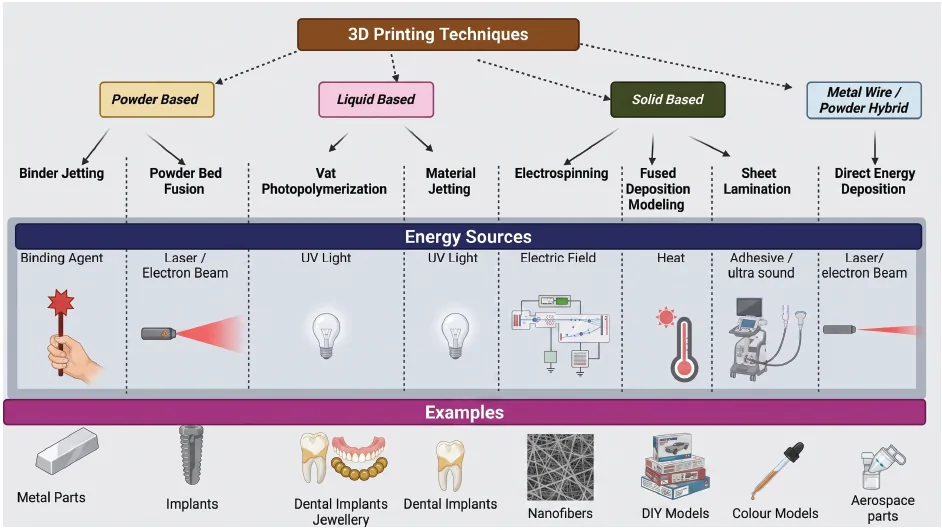
Classification of 3D printing techniques based on material type and energy source. Representative applications span from implants and models to advanced aerospace components.

**Fig. (2):**
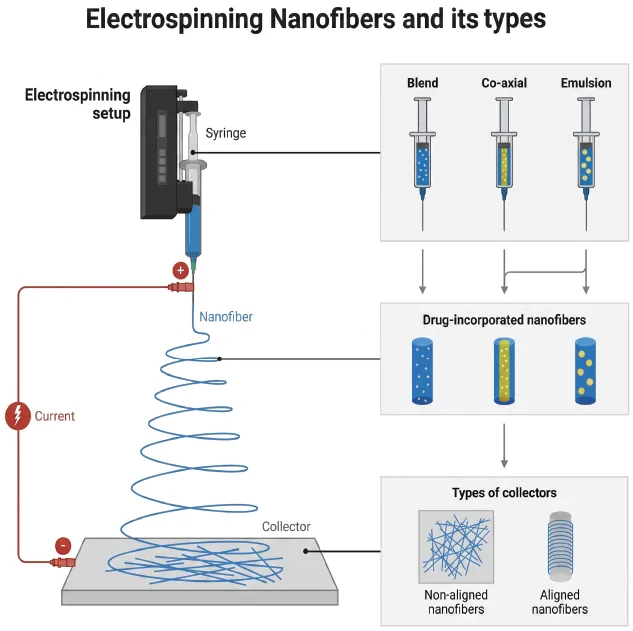
Schematic of electrospinning setup and types of nanofibers produced. Different configurations enable drug incorporation and fiber alignment for targeted application.

**Fig. (3):**
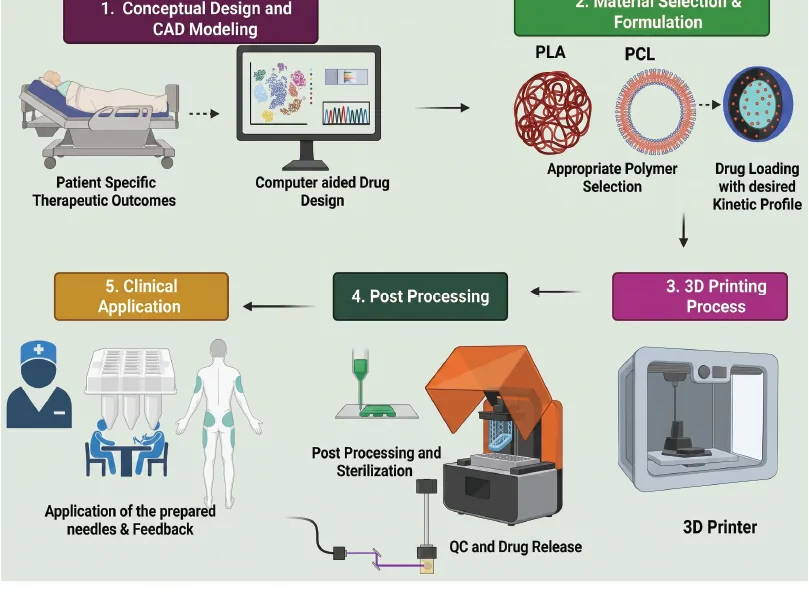
A simplified workflow of 3D-printed drug delivery systems, illustrating the steps from CAD design and material selection to printing, post-processing, and personalized clinical application.

**Fig. (4):**
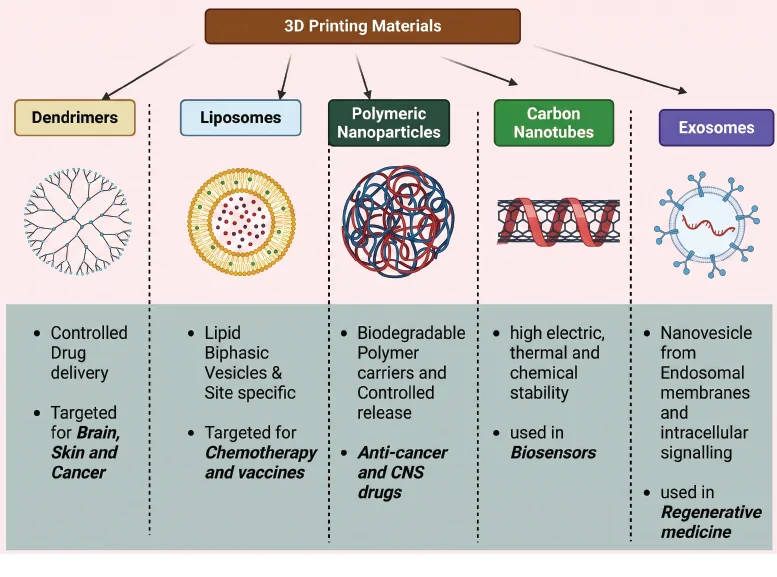
Overview of nanomaterials used in 3D printing for drug delivery applications. Each material type is associated with specific therapeutic targets and biomedical functionalities.

**Table 1:** Summary of advanced nanocarriers used in drug delivery systems.

**Nanocarrier Method**	**Drug Encapsulated**	**Modification Material**	**Outcome**	**References**
Nanotube	Phospholipid nanotube nanodrills	Human Serum Albumin	Longer nanodrills enhance intracellular uptake efficiency and penetrate the extracellular matrix, demonstrating tissue-penetrating capacity.	[94]
Niosomes	Ascorbic acid & Curcumin	Dual-drug-loaded Niosomal (Asc + Cur)	The co-encapsulation of Cur and Asc in vesicles enhanced anticancer properties and sustained release rates. The nanoniosomes showed 70% enhanced cellular cytotoxicity and higher levels of oxidative cell damage.	[95]
Lipid nanoparticle	DLin-MC3-DMA (MC3) and SM-102	β-catenin mRNA	The two FDA-approved ionizable phospholipids, DLin-MC3-DMA (MC3) and SM-102, were used to produce specialized ionizable Lipid Nanoparticles (LNPs). SM-102 LNPs demonstrated superior delivery of genetic material and prolonged effectiveness compared to MC3 LNPs. Moreover, SM-102 LNPs showed minimal disruption from fractures and no localized inflammatory reaction when tested *in vivo* models.	[96]
Nanoparticles Chitosan Quaternary Ammonium Salt (CQAS), Amine functionalized Silica Nano Powder (ASNP), and Carbon Quantum Dots (CQD)	1. ASNP-dsRNA NPs 2. CQAS-dsRNA NPs 3. CQD dsRNA NPs	NPs in RNAi	All three types of NPs effectively silenced genes and provided at least 14 days of protection against viral infection. CQD-dsRNA NPs could protect systemic leaves for 21 days following spraying.	[97]
Carrier-free self-assembly nanomedicine	Curcumin & icariin (ICA) nanoparticle	Fluorescent tracer Cur /ICA NP	The nanoparticles exhibit low cytotoxicity, high cellular uptake, and sustained drug release, thereby inhibiting inflammatory cytokines and reducing cartilage degeneration. They also exhibit superior anti-inflammatory and cartilage protection effects.	[98]
cationic soybean oil-based polyurethane ionene nanocarrier	5-fluorouracil (5FU)	folic acid	A new CPUI nanocarrier loaded with 5-fluorouracil and coated with folic acid has been developed for the targeted delivery of cancer drugs. *In vitro* and *in vivo* experiments show that nanocarriers effectively inhibit tumor growth, potentially benefiting targeted intracellular drug delivery.	[99]
Natural polymers, alginate and chitosan, into a polyelectrolytic nano-formulation.	Diallyl disulfide	Nanoencapsulation of Diallyl disulfide	Nanoencapsulation of Diallyl disulfide with natural polymers, such as alginate and chitosan, can increase its bioavailability, specificity, and efficacy. It also efficiently downregulated p38 mitogen-activated protein kinase, thereby reducing pro-inflammatory protein expression.	[100, 101]
Reverse micelle (RM)-based lipocarriers	vascular endothelial growth factor-C	Fabricated an engineered VEGF-C protein using recombinant human VEGF-C protein (rhVEGF-C)	Nanolipocarriers with LV-specific VEGF-C could improve lymphatic drainage, gut immunity, and portal hypertension, potentially opening new avenues for treating and preventing decompensation in cirrhosis.	[101]
Bee pollen extract (BPE) based polymer nanoparticles	Avastin	Combination of bee pollen extract and Avastin	Bee pollen extract and its prepared nanoparticles demonstrated potent anticancer efficacy against A549 lung cancer cells, with high flavonoid and polyphenol content having the most profound effect. Combining BPENPs with Avastin chemotherapy showed a novel synergism, affecting HRAS, MAPK pathways, and apoptotic genes.	[102]
Alginate chitosan nanoparticles	Madecassoside	Drug encapsulated in nanoparticles	MACNPs reduced severe seizures and improved bioavailability and membrane integrity.	[103]
Nanosuspension	Metronidazole	Metronidazole nanosuspension loaded into microarray patches	MTZ-NS showed minimal toxicity and biocompatibility. The formulation has a 95% delivery efficiency after 24 hours and complete bacterial inhibition.	[104]

**Note:** This table highlights various nanocarriers, encapsulated drugs, and their biomedical outcomes. Each formulation demonstrates enhanced delivery, targeting, or therapeutic efficacy in preclinical studies.

## LIST OF ABBREVIATIONS

AM: Additive Manufacturing
CAD: Computer-Aided Design
DED: Direct Energy Deposition
DMLS: Direct Metal Laser Sintering
EBM: Electron Beam Melting
FDM: Fused Deposition Modeling
LENS: Laser-Engineered Net Shaping
RP: Rapid Prototyping
SHS: Selective Heat Sintering
SLA: Stereolithography
SLS: Selective Laser Sintering
